# Regulation of DCC Localization by HTZ-1/H2A.Z and DPY-30 Does not Correlate with H3K4 Methylation Levels

**DOI:** 10.1371/journal.pone.0025973

**Published:** 2011-10-05

**Authors:** Emily Petty, Emily Laughlin, Györgyi Csankovszki

**Affiliations:** Department of Molecular, Cellular, and Developmental Biology, University of Michigan, Ann Arbor, Michigan, United States of America; Florida State University, United States of America

## Abstract

Dosage compensation is a specialized form of gene regulation that balances sex-chromosome linked gene expression between the sexes. In *C. elegans*, dosage compensation is achieved by the activity of the dosage compensation complex (DCC). The DCC binds along both X chromosomes in hermaphrodites to down-regulate gene expression by half, limiting X-linked gene products to levels produced in XO males. Sequence motifs enriched on the X chromosome play an important role in targeting the DCC to the X. However, these motifs are not strictly X-specific and therefore other factors, such as the chromatin environment of the X chromosome, are likely to aid in DCC targeting. Previously, we found that loss of HTZ-1 results in partial disruption of dosage compensation localization to the X chromosomes. We wanted to know whether other chromatin components coordinated with HTZ-1 to regulate DCC localization. One candidate is DPY-30, a protein known to play a role in DCC localization. DPY-30 homologs in yeast, flies, and mammals are highly conserved members of histone H3 lysine 4 (H3K4) methyltransferase Set1/MLL complexes. Therefore, we investigated the hypothesis that the dosage compensation function of DPY-30 involves H3K4 methylation. We found that in *dpy-30* animals the DCC fails to stably bind chromatin. Interestingly, of all the *C. elegans* homologs of Set1/MLL complex subunits, only DPY-30 is required for stable DCC binding to chromatin. Additionally, loss of H3K4 methylation does not enhance DCC mislocalization in *htz-1* animals. We conclude that DPY-30 and HTZ-1 have unique functions in DCC localization, both of which are largely independent of H3K4 methylation.

## Introduction

The intricate regulation of gene expression that occurs in all organisms is the result of the coordinated activity of multiple machineries (RNA Polymerases, transcription factors, histone modifiers, chromatin modifiers, etc.). In order for this to be achieved, these machineries need to be targeted to very specific sites of action within the vast expanse of the genome. How these various proteins and complexes find their proper targets is an important question to answer to further our understanding of how gene regulation is ultimately achieved.

Here we discuss the targeting of a specialized gene regulatory complex in *C. elegans* called the dosage compensation complex (DCC). In organisms such as *C. elegans*, the difference in sex chromosome number between the sexes, if left uncorrected, would result in an imbalance of X-linked gene expression in the affected sex. To correct this imbalance, the DCC is recruited to the two X chromosomes in *C. elegans* hermaphrodites and dosage compensated genes are down-regulated an average of two-fold to match expression levels observed in the XO male (reviewed in [1], [2], [3]). The DCC is comprised of a regulatory subcomplex composed of SDC-1, SDC-2, SDC-3, DPY-30, and DPY-21, and an enzymatic condensin complex comprised of DPY-27, MIX-1, CAPG-1, DPY-28, and DPY-26 (condensin I^DC^), similar to mitotic and meiotic condensin complexes [1], [4]. SDC-2 and SDC-3 can bind to chromatin independent of the condensin complex but the reverse is not true [5], [6], [7].

Although the gene regulation function of dosage compensation in flies is opposite of worms, there are similarities in the X-chromosome targeting mechanisms employed in the two species. In *D. melanogaster*, dosage compensation is achieved by up-regulation of the single male X chromosome by the MSL (*m*ale-*s*pecific *l*ethal) complex [8], [9]. The initial targeting of the MSL complex to the X chromosome occurs at chromatin entry sites or high affinity sites containing GA-dinucleotide repeats [10], [11]. The entry sites are also specifically depleted of nucleosomes [10]. The MSL complex then spreads locally onto the 3′ ends of nearby active genes [12], [13], [14]. The complex can identify active genes by the specific recognition of H3K36 methylation at the 3′ end of genes by the subunit MSL3 [15]. Therefore, sequence information, transcriptional status, and chromatin organization play key roles in recruitment and spreading of the MSL complex in fly dosage compensation [10], [11], [12], [13], [14], [15], [16].

DCC binding in *C. elegans* also involves a similar combination of factors. Analysis of DCC binding by chromatin immunoprecipitation, as well as analysis of DCC binding to transgene arrays, revealed the presence of multiple classes of DCC binding sites [17], [18], [19], [20], [21]. Regions that can attract DCC binding independently are called *rex* sites (for *r*ecruitment *e*lement on *X*) and all other sites of DCC binding are called *dox* sites (for *d*ependent *o*n *X*) [7], [18], [20]. We found at least one other class of DCC binding sites that function as *rex* sites on multicopy transgenic arrays, but fail to attract robust complex binding in single copy chromosomal duplications [21]. We refer to such sites as way-stations and predict that these sites assist in DCC spreading downstream of recruitment. Motif searches of both recruiting elements and regions with exceptionally high DCC occupancy led to the identification of a 12 bp consensus sequence named MEX, for *M*otif *E*nriched on *X* [17], [18], [20]. Regions of the X containing MEX are able to recruit the DCC *in vivo*, indicating that the sequence has recruitment function. However, neither the presence of MEX nor high levels of DCC binding strictly correlate with the ability to recruit the DCC [17], [20], [21], indicating that MEX is not the sole driver of DCC recruitment.

Evidence suggests that transcriptional activity may also contribute to DCC targeting. The DCC binds to promoter regions of about half of all transcribed genes on X [17], [18], [22]. In an X:A chromosomal fusion strain, binding to promoters of active genes on the attached autosome is augmented. Furthermore, changes in gene expression during development correlate with changes in DCC binding [7], [22]. These results indicate that transcriptional activity may help attract the DCC independent of chromosome-specific sequences [22]. Curiously, DCC-mediated gene expression changes do not correlate with the presence of DCC at the promoter [18].

Chromatin organization also likely plays an important and as yet incompletely understood role in *C. elegans* dosage compensation. Indeed, previously we have shown that the H2A.Z variant in *C. elegans* (HTZ-1) plays a role in regulating DCC targeting to the X chromosomes [23]. H2A.Z is a histone variant conserved in all eukaryotes and is essential in all organisms tested except yeast [24], [25], [26]. It is highly associated with promoter regions, however this is not necessarily associated with active expression [27], [28], [29], [30], [31], [32]. Although not fully understood, H2A.Z likely functions to positively regulate gene expression [33], [34], [35], [36], [37], [38], [39]. H2A.Z displays antagonistic activity with repressive chromatin complexes and signatures [23], [40], [41], [42], [43], [44]. In yeast, Htz1 is required to restrict the spread of silencing complexes from the silent mating type loci and telomeres [43], [44], [45]. In *Arabidopsis* and human cells, H2A.Z specifically antagonizes DNA methylation, a signature of long-term gene silencing [40], [42].

In *C. elegans,* HTZ-1 is found at significantly lower levels on the X chromosomes than autosomes [23], [29]. Depletion of HTZ-1 in hermaphrodites leads to ectopic DCC localization and disrupts dosage compensation [23]. Therefore, we hypothesized that HTZ-1 functions to prevent autosomal binding of the DCC in hermaphrodites, similar to the functions described in yeast, *Arabidopsis*, and humans, in antagonizing the spread of gene repression activities.

In search of other chromatin factors regulating DCC binding, we decided to investigate the role of H3K4 methylation. In budding yeast, Htz-1 cooperates with H3K4 methylation to restrict genome-wide spreading of the SIR (*S*ilent *I*nformation *R*egulator) complex [44]. Additionally, loss of H3K4 methylation by disruption of Set1 complex function results in increased heterochromatin signatures (H3K27 and H3K9 methylation) at chicken β-globin insulator elements [46]. H3K4 methylation is the result of the function of Set1/MLL histone methyltransferase complexes made up of a SET-domain containing methyltransferase (of the Set1 or MLL family) and at least four other subunits, WDR5, RbBP5, Ash2L, and DPY-30 [47], [48], [49], [50], [51], [52], [53], [54], [55], [56]. Intriguingly, orthologs of the known dosage compensation protein, DPY-30, are found in all Set1/MLL complexes and contribute to methylation activity of the complexes in other organisms [50], [51], [56], [57], [58], [59]. DPY-30 binds ASH2L, and also forms homodimers, allowing for dimerization of the entire complex [58], [59]. Without DPY-30, global levels of H3K4me3 are significantly reduced in budding yeast, *C. elegans*, and mammals [7], [57], [58], [59], [60], [61]. Slight reductions of dimethylation have also been reported in budding yeast and *C.elegans* [58], [60].

In *C. elegans*, in addition to a global reduction in H3K4 methylation, *dpy-30* mutant hermaphrodites exhibit DCC mislocalization and impaired dosage compensation [7], [62], [63]. Recent data suggest that DPY-30 function is important for DCC recruitment in a similar fashion as the recruitment proteins SDC-2 and SDC-3 [7]. Interestingly, the *C. elegans* Ash2L homolog, ASH-2, can also be found at many recognition sites and overlaps DCC binding at some genes [7]. Overall, current evidence suggests that DPY-30 is a member of both DCC and Set1/MLL complexes. However, it has not been tested whether DPY-30 function in histone methyltransferase complexes, or H3K4 methylation itself, contributes to dosage compensation. It has recently been proposed that DPY-30 and H3K4 methylation may assist in DCC spreading to sites of active transcription [2].

We decided to test whether HTZ-1 and H3K4 methylation cooperate in regulating DCC binding, so that in concert with sequence- and transcription-driven targeting, the DCC is targeted correctly to the X chromosomes. We observed DCC localization in Set1/MLL depleted animals and in *htz-1* mutants depleted of Set1/MLL subunits. Depletion of these components led to decreased H3K4me3, as expected. However, we did not observe a correlation between H3K4me3 reduction and effects on DCC localization. Furthermore, DCC mislocalization in *htz-1* animals was not enhanced by reduced H3K4 methylation. We conclude that H3K4 methylation does not signifcantly contribute to HTZ-1-regulated DCC localization and that the function of DPY-30 in DCC localization is largely independent of its role in Set1/MLL histone methytransferase complexes.

## Results

### H3K4 is differentially methylated on the dosage compensated X chromosomes

In order to investigate whether H3K4 methylation regulates DCC localization we first observed the methylation status of H3K4 on the dosage compensated X chromosomes at the resolution of a single cell using immunofluorescence (IF) analysis in adult hermaphrodite intestinal nuclei. We utilized antibodies specific for the mono-, di-, and trimethylated forms of H3K4 along with antibodies specific for subunits of the dosage compensation complex (DCC) to label the X chromosomes. To compare intensity of the methylated H3K4 species on the X chromosomes to the rest of the nucleus, we measured the ratio of the average H3K4me signal intensity in the DCC positive region compared to the average intensity of the entire nucleus (see Materials and Methods). We compared these results to measurements of HTZ-1 intensity on X which has been previously shown to be depleted on the X chromosomes in hermaphrodite animals [23], [29].

**Figure 1:**
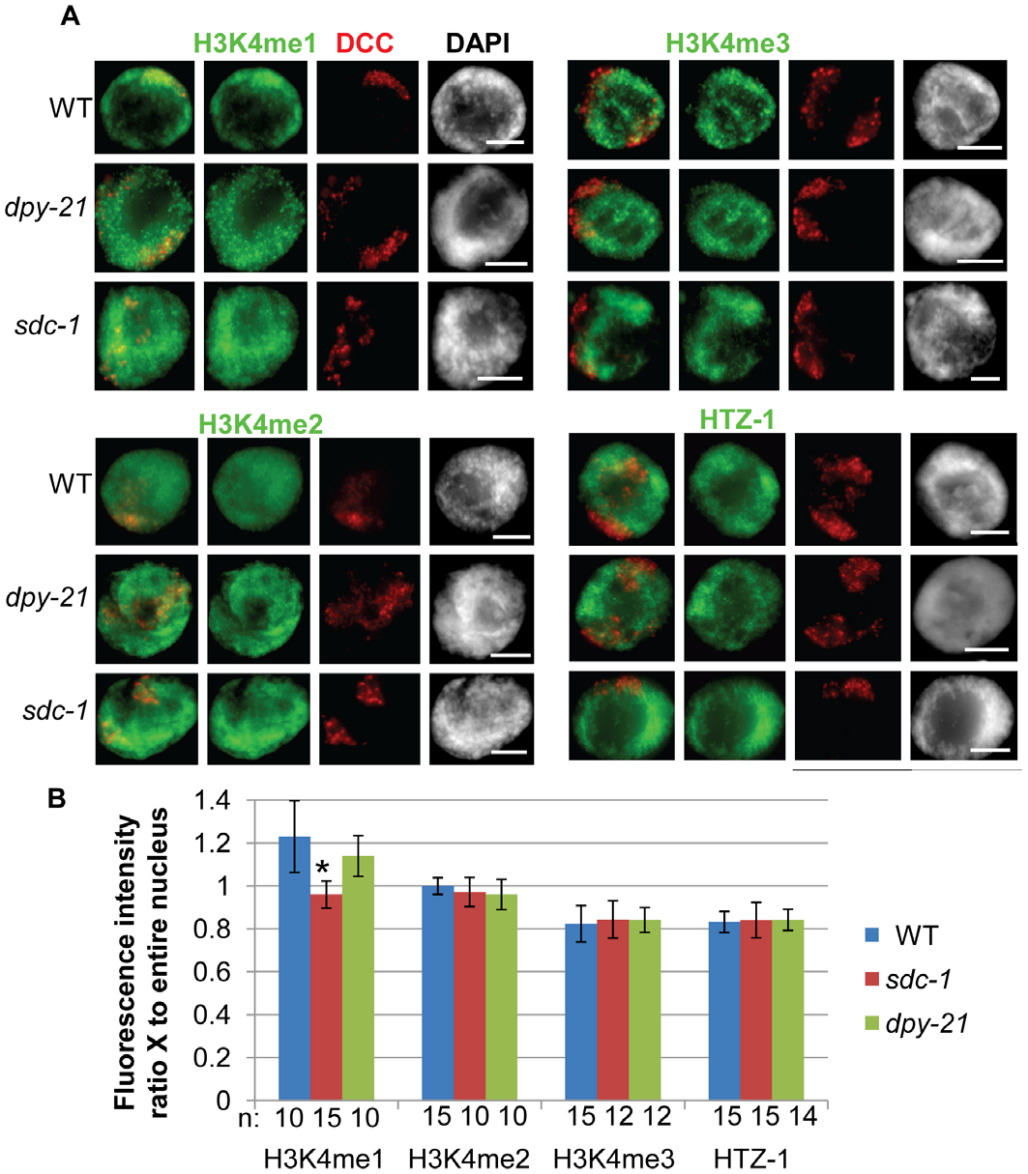
H3K4 is differentially methylated on dosage compensated X chromosomes. **A.** IF of the DCC (red) and H3K4me1, H3K4me2, H3K4me3 or HTZ-1 in wildtype, or dosage compensation mutants *sdc-1(e415*) and *dpy-21*(*e428*) shows slightly higher levels of H3K4me1 and lower levels of both H3K4me3 and HTZ-1 on dosage compensated X chromosomes in adult hermaphrodite intestinal nuclei. DCC is marked by a DPY-27-specific antibody (with H3K4me2, H3K4me3 and HTZ-1 co-stain) or a CAPG-1-specific antibody (with H3K4me1 co-stain). DAPI (grayscale) stains DNA. Scale bar equals 5 µm. **B.** Quantification of H3K4 methylation and HTZ-1 occupancy on the dosage compensated X chromosomes by IF in wildtype and dosage compensation mutants. The average intensity of H3K4 methylation or HTZ-1 signal was recorded for the nucleus and for the DCC-marked X chromosome regions. The ratio of the average intensity on X versus the average intensity for the entire nucleus was calculated and the average values are shown. Error bars indicate the standard deviation. Star indicates significant difference from wildtype by t-test analysis (p = 6×10^−3^).

Interestingly, we found the average H3K4me3 intensity signal to be reduced on the X by nearly 20% compared to the average signal in the nucleus. This is almost identical to the reduction observed for HTZ-1 signal intensity on X. H3K4me2 signal intensity is the same, while the H3K4me1 signal is slightly higher on the X chromosome compared to the rest of the nucleus (Fig. 1). These results are consistent with the recent genome-wide ChIP-chip results in larval L3 animals, where the greatest difference in H3K4 methylation on X was observed with H3K4me3 [64].

### H3K4 differential methylation is not regulated by dosage compensation

We next wanted to investigate whether the differences in methylation levels on the X chromosomes are regulated by dosage compensation. To do this we performed the same analysis as described above in the dosage compensation mutants *sdc-1(y415)* and *dpy-21(e428)*. We chose these specific mutants because although dosage compensation function is disrupted by these mutations, the DCC still localizes to the X chromosome [65]. This allows us to again utilize DCC specific antibodies to label the X. The alternative method of labeling the X chromosome by X Paint fluorescent *in situ* hybridization (FISH) was not possible as the H3K4 methylation antibodies fail when combined with FISH.

For the most part, the differential methylation and differential HTZ-1 levels on the X were unchanged in dosage compensation mutants (Fig. 1). The only exception was in *sdc-1* where the slight enrichment of H3K4 monomethylation was lost. These results indicate that differential H3K4me3 and HTZ-1 levels on the X chromosome are not regulated by dosage compensation. The loss of H3K4me1 enrichment in *sdc-1* indicates that dosage compensation may be responsible for monomethylation enrichment on the X chromosomes. However, the fact that enrichment is still observed in *dpy-21* mutants indicates that there is no strict correlation between loss of enrichment and loss of dosage compensation function.

### DPY-30 is required for DCC localization and binding to chromatin

Next, we wanted to understand the effect of the Set1/MLL component DPY-30 on DCC localization at the resolution of a single cell. Previously, DPY-30, a subunit of Set1/MLL, was described to cause DCC mislocalization [63] and very recently was shown to be required for DCC recruitment to the X by ChIP-chip analysis in mixed stage embryo preparations [7]. Whether this phenotype is due to changes in H3K4 methylation has not been addressed. We utilized DPY-27 IF combined with X-Paint FISH to observe the extent of DCC mislocalization in homozygous *dpy-30(y228)* mutant animals decended from heterozygous mothers (m+z). DPY-30 is maternally provided to these progeny, but the zygote does not produce fully functional protein.

**Figure 2:**
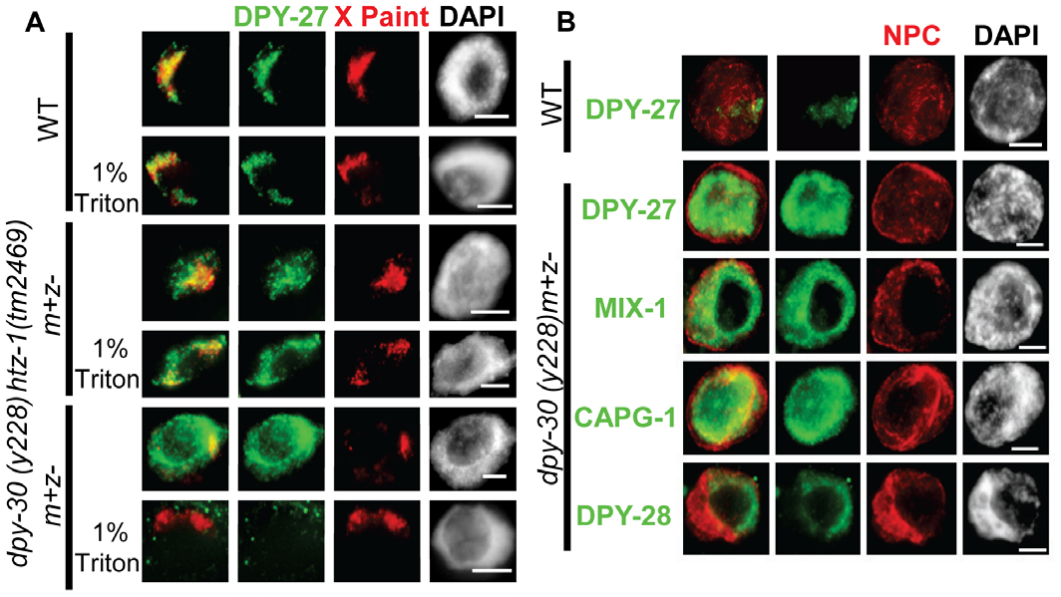
DCC binding to or stability on chromatin is compromised in *dpy-30* animals. **A.** DCC localization by DPY-27 IF (green) combined with X-paint FISH (red) with and without detergent extraction of nucleoplasmic contents in wildtype, *dpy-30(y228*), and *htz-1*(*tm2469*) hermaphrodite intestinal nuclei shows that the diffuse DCC localization observed in *dpy-30* animals can be extracted with detergent treatment while the mislocalized DCC in *htz-1* cannot. **B.** Condensin I^DC^ components mislocalize in *dpy-30(y228)* hermaphrodite animals. DPY-27, DPY-28, CAPG-1, and MIX-1 (green) all show the same diffuse, nuclear pattern in hermaphrodite adult intestinal nuclei. DPY-27 localization in wildtype (WT) is shown as a control. The nuclear envelope is marked with a nuclear pore complex antibody (red) as a staining control. In all panels DAPI is shown in grayscale and scale bar equals 5 µm.

In *dpy-30* nuclei, DPY-27 has a completely diffuse, nuclear appearance with inconsistent overlap with the X chromosomes (Fig. 2A). The signal is so diffuse that we hypothesized that the DCC was not bound to chromatin, but rather largely nucleoplasmic. To test this we performed a detergent extraction prior to fixing the tissue to remove nucleoplasmic content. What we observed was striking- most of the DPY-27 signal was lost upon extraction. This strongly suggests that DPY-30 is required for the complex to stably bind chromatin. These results are in contrast to what we observe in *htz-1(tm2469).* As reported previously, *htz-1* animals exhibit ectopic DCC localization to autosomes and this ectopic signal is not sensitive to detergent extraction (Fig. 2A and [23]. Therefore, while both DPY-30 and HTZ-1 affect DCC localization, they do so in a markedly different manner.

Previously, it was shown that DCC assembly on the X chromosome required all subunits such that the absence of one component affected the stability or localization of the rest of the complex [4], [66], [67], [68], [69]. Because of this, we hypothesized that the DCC as a whole was mislocalized in *dpy-30* animals, rather than DPY-27 on its own. To address this we observed three other members of the complex, MIX-1, CAPG-1, and DPY-28 by IF in *dpy-30* nuclei. All of these components had a diffuse, nuclear pattern in *dpy-30* exactly as we observe for DPY-27 (Fig. 2B). This result strongly suggests that the DCC as a whole cannot load onto chromatin in *dpy-30* animals.

### Set1/MLL depletion affects H3K4 methylation, but not DCC localization

We next wanted to test whether the loss of Set1/MLL subunits other than DPY-30 has a similar effect on DCC localization. We hypothesized that the role of DPY-30 in dosage compensation was linked with its function in the histone methyltransferase complexes and predicted that loss of other Set1/MLL subunits would also disrupt DCC binding to chromatin. To test this, we depleted individual members of the Set1 and MLL complexes by RNAi and observed DCC localization in the adult animals. Although we tested all homologs by RNAi, we focused on the two SET-domain containing genes *set-2* (homologous to Set1 in yeast, Set1A in humans) and *set-16* (homologous to MLL2/3 in mammals) as well as *ash-2* (homologous to Bre2 in yeast and Ash2L in humans) and *hcf-1* (homologous to human Hcf1).

We focused on *set-2* and *set-16* as these are the only genes in *C. elegans* homologous to H3K4 methyltransferases in other organisms and have been shown previously to function in H3K4 methylation [60], [61], [70], [71]. We focused on *ash-2* because the homologs in other organisms are known binding partners of DPY-30 in Set1/MLL complexes and this interaction is essential for full methyltranferase activity [57], [58], [59]. Finally, we chose *hcf-*1 because the mammalian homologs, in addition to being a physical component of Set1/MLL, serve as molecular tethers between MLL and other chromatin modifying complexes [54], [72], [73]. To observe the effect of individual subunit depletion on Set1/MLL complex activity we analyzed H3K4me3 levels by IF and western blot as loss of individual components in other systems affects this modified form most dramatically [49], [50], [58], [59], [74]. We also analyzed H3K4me2 and H3K4me1 levels by IF and western blot upon depletion of SET-2, SET-16, ASH-2, HCF-1, and DPY-30.

**Figure 3:**
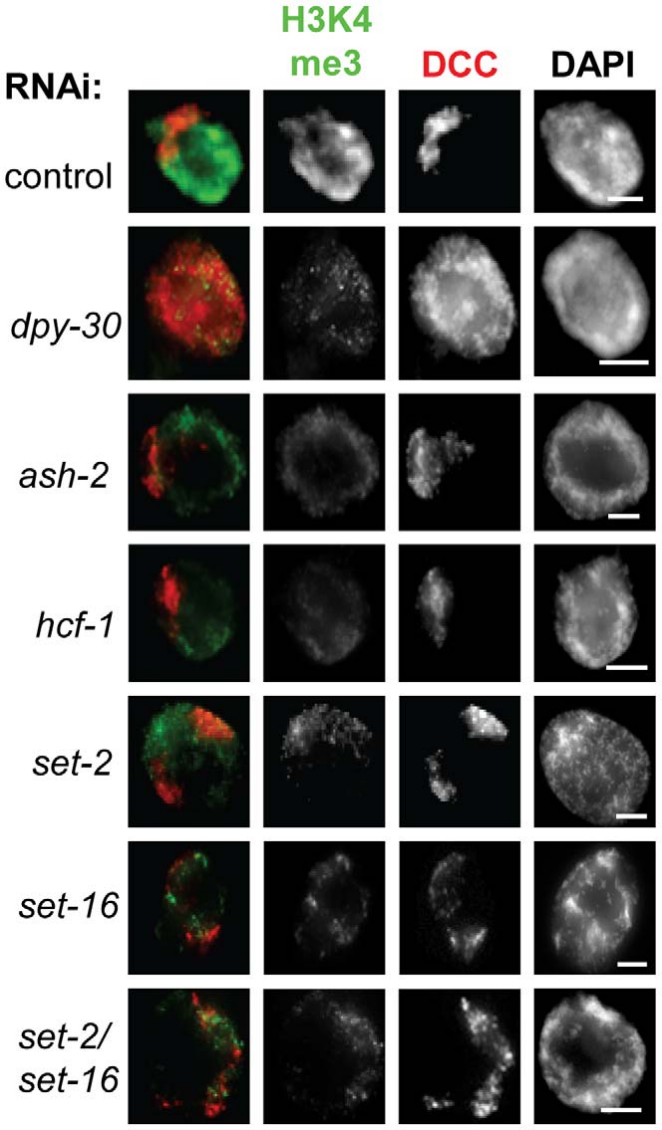
Depletion of Set1/MLL subunits by RNAi leads to reduction in H3K4me3 levels. H3K4me3 (green) and DPY-27 (red) IF in control RNAi and Set1/MLL depletion animals shows a reduced H3K4me3 signal upon Set1/MLL RNAi in adult intestinal nuclei. Note that only DPY-30 depletion leads to a diffuse DCC localization phenotype. In all panels DAPI is shown in grayscale and scale bar equals 5 µm.

We found that depletion of each individual Set1/MLL subunit homolog led to a reduction in H3K4me3 (Fig. 3 and Fig. S1). This is consistent with the fact that full H3K4 methyltransferase activity by the Set1/MLL complex requires all subunits in yeast and mammals [49], [57], [58], [59], [74], [75], [76], [77], [78]. By contrast, mono and di-methylation signals were less affected (Fig. S2). Only depletion of the catalytic subunits SET-2 and SET-16 reduced H3K4me2 and H3K4me1 signals. Depletion of ASH-2 leads to a reduction in di- and trimethylation, consistent with published *in vivo* analysis in budding yeast and mammals [58], [59], [74], [76], [78]. In mammals, heterodimers of Ash2L and RbBP5 exhibit SET-independent monomethylation activity *in vitro* [79], however, we do not observe any change in H3K4 monomethylation levels in ASH-2 depleted animals. DPY-30 and HCF-1 depletion animals showed reduced H3K4me3 by IF, but di- and monomethylation signals were unchanged (Fig. S2). As a control, we also analyzed H3K27me3 signal by IF in Set1/MLL depletion and mutant animals and found no striking change in H3K27me3 signal, as expected (Fig. S3). Western blot analysis confirmed what was observed by IF: SET-2 and SET-16 depletion leads to reductions in all forms of H3K4 methylation, ASH-2 depletion affects di- and trimethylation, and DPY-30 depletion specifically affects H3K4 trimethylation in young adult hermaphrodites (Fig. S4). Analysis of *set-2(ok952)* and *hcf-1(ok559)*, both hypomorph deletion mutants [80], [81], as well as *set-2(bn129)* and *wdr-5.1(ok1417)*, both null deletion mutants [60], [81], led to similar conclusions. The decrease in methylation levels in the *set-2(ok952)* and *hcf-1(ok559)* mutants was less severe than after RNAi depletion (Fig. S5). Analysis of H3K4 methylation in both the *set-2(bn129)* as well as a *wdr-5.1(ok1417)* null strains revealed stronger effects on H3K4me3 and H3K4me2 in particular (Fig. S5).

Surprisingly, the only Set1/MLL component that also affects DCC localization is *dpy-30*. Depletion of DPY-30 by RNAi leads to the same diffuse, nuclear DCC localization pattern as we observe in the *dpy-30* mutant. In all other Set1/MLL depletion animals, the level of H3K4me3 staining was similar to DPY-30 depletion animals, but the DCC staining pattern appeared exactly as in controls (Fig. 3, Fig. S1). The results of depletion of the individual Set1/MLL subunits along with the names of their respective *S. cerevisiae* and human homologs are summarized in Table 1.

**Table 1:** Summary of Set1/MLL depletion.

*S. cerevisiae*	Human	*C. elegans*	H3K4me3	DCC
*SET1*	*hSET1*	*set-2*	reduced	WT
	*MLL2/3*	*set-16*	reduced	WT
*SWD1/Cps50*	*RbBP5*	F21H12.1	reduced	WT
*SWD3/Cps30*	*WDR5*	*wdr-5.1*	reduced	WT
		*swd-3.2*	reduced	WT
		*swd-3.3*	reduced	WT
*SWD2/Cps35*	*WDR82*	*swd-2.1*	reduced	WT
		*swd-2.2*	reduced	WT
	*WDR83*	F33G12.2	reduced	WT
*SPP1/Cps40*	*CFP1*	F52B11.1	reduced	WT
*BRE2/Cps60*	*Ash2L*	*ash-2*	reduced	WT
*SDC1/Cps25*	*hDPY30*	*dpy-30*	reduced	Diffuse
	*HCF1/2*	*hcf-1*	reduced	WT

The Set1/MLL homologs tested in this study are shown. Names of budding yeast and mammalian homologs are included for reference. Depletion of each subunit led to reduced H3K4me3 by IF or a combination of IF and western. Only *dpy-30* depletion resulted in diffuse DCC localization. In all other Set1/MLL depletion animals, DCC localization appeared wild-type (WT).

**Figure 4:**
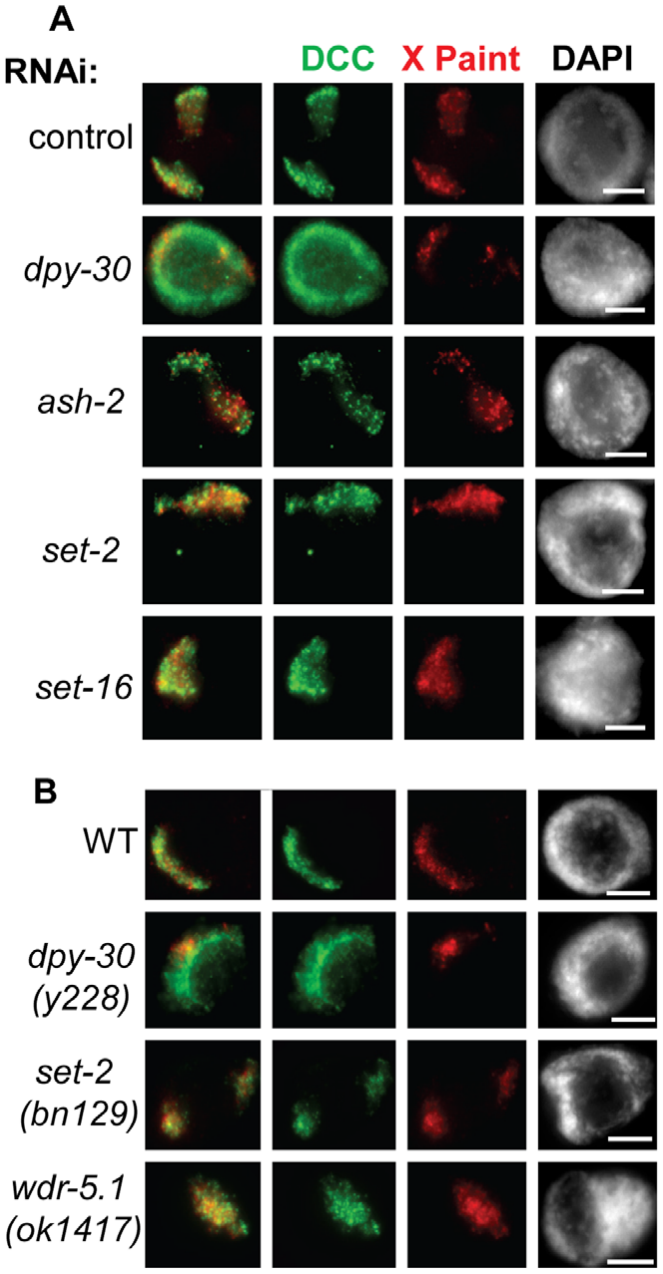
DCC localization to the X chromosomes is disrupted in *dpy-30* depleted and mutant animals but not in other Set1/MLL depletion and mutant animals. DPY-27 (green) and X-Paint FISH (red) in control and Set1/MLL depletion (**A**) and mutant (**B**) animals. In all panels DAPI is shown in grayscale and scale bar equals 5 µm.

Although the DCC appears normal in all of the Set1/MLL depletion animals except *dpy-30*, we wanted to be certain that the DCC was truly localizing to the X chromosomes. Again, we combined DCC IF with X-Paint FISH to observe DCC localization on the X chromosomes in Set1/MLL depletion animals. We found that the DCC signal colocalized with the X-Paint signal in the Set1/MLL depletion animals similar to the control (Fig. 4A). Additionally, the two null strains, *set-1(bn129)* and *wdr-5.1(ok1417)*, showed no significant change in DCC localization compared to wildtype hermaphrodites, further demonstrating that loss of these components does not significantly affect DCC localization (Fig. 4B).

**Figure 5:**
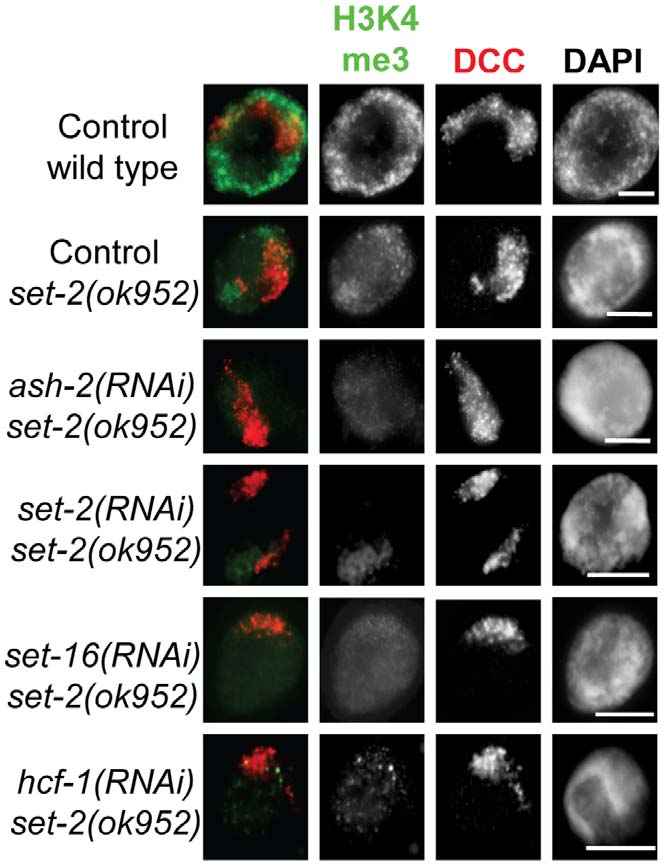
Severe loss of H3K4 methylation by Set1/MLL depletion in *set-2(ok952)* hermaphrodites. Hermaphrodite intestinal nuclei co-stained for H3K4me3 (green) and DPY-27 (red) in Set1/MLL complex depletion in *set-2* mutant animals demonstrates that increased loss of H3K4 methylation does not correlate with DCC mislocalization. In all panels DAPI is shown in grayscale and scale bar equals 5 µm.

We next hypothesized that residual Set1/MLL activity remaining upon depletion of only one component prevented us from observing a change in DCC localization. To address this, we performed RNAi depletion of SET-2, SET-16, ASH-2, and HCF-1 in the *set-2(ok952)* deletion mutant background. We observed further reductions of H3K4me3 (Fig. 5), H3K4me2, and H3K4me1 signals (Fig. S6). We still did not observe any obvious change in DCC localization in these animals. This result suggests that Set1/MLL complexes and H3K4 methylation are not involved in dosage compensation at the level of DCC localization and stability on chromatin. These data also indicate that DPY-30's role in DCC localization is likely to be independent of its function in H3K4 methylation.

### Set1/MLL complexes do not cooperate with *htz-1* to restrict DCC localization

Although neither Set1 nor MLL complexes appear to play a role in DCC localization or binding to chromatin on their own, it is possible that these complexes may function with other chromatin components to affect DCC localization. Previous work in budding yeast has demonstrated that Set1C cooperates with the histone variant H2A.Z/Htz1 to limit genome-wide mislocalization of the SIR complexes [44]. Our lab has shown that loss of HTZ-1 in *C. elegans* results in partial mislocalization of the DCC to autosomes [23]. We therefore wanted to test whether H3K4 methylation defects enhance DCC mislocalization in *htz-1* animals. To do this we depleted Set1/MLL components in the *htz-1(tm2469)m+z-* background. We then used DCC IF combined with X-Paint FISH to visualize DCC localization to the X chromosomes at the single cell resolution. To measure the degree of DCC mislocalization we measured colocalization of the DCC and X-Paint signals using Slidebook software. The result of this analysis is a Pearson's correlation value (*R*) where 1 represents complete colocalization of the signals and 0 represents no measureable colocalization [23].

**Figure 6:**
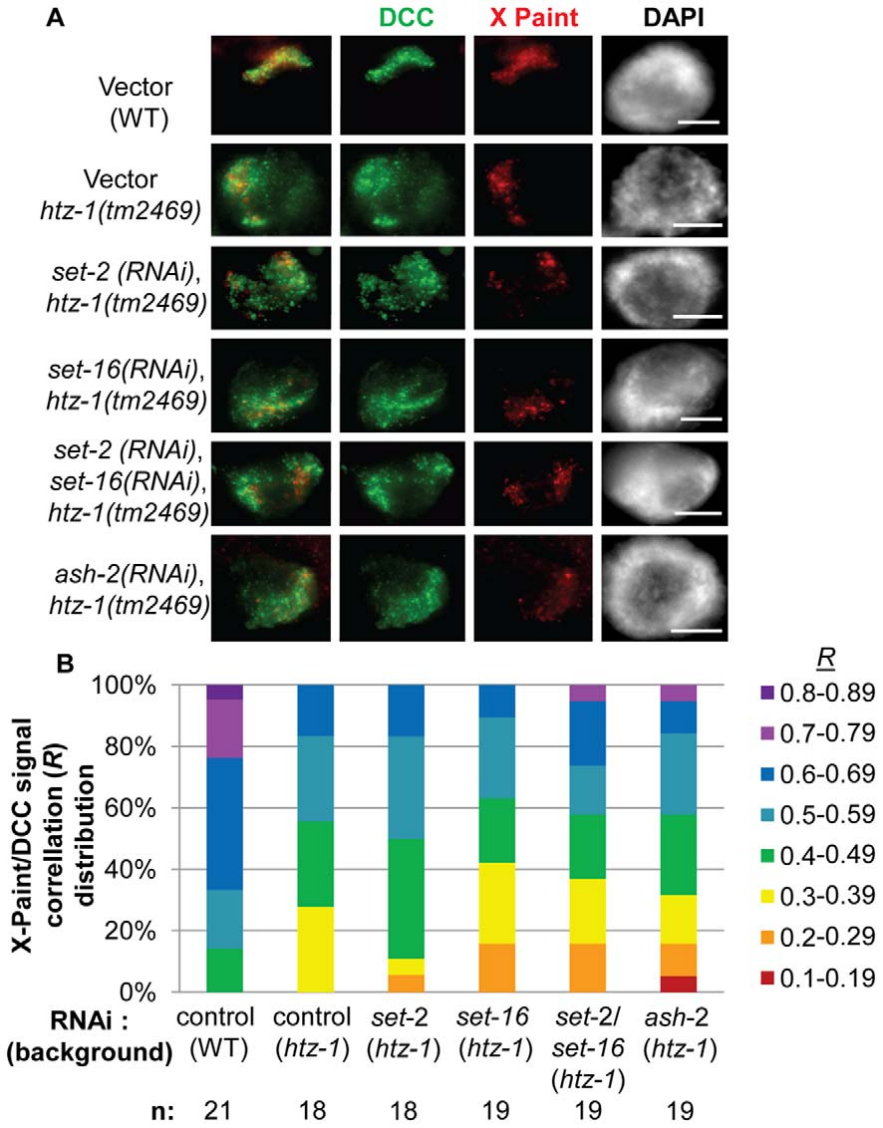
Reduced H3K4 methylation by Set1/MLL depletion does not enhance DCC mislocalization in *htz-1(tm2469)*. DPY-27 (green) and X-Paint FISH (red) colocalization analysis upon Set1/MLL depletion in *htz-1(tm2469)m+z-* mutant animals indicates that reduced H3K4 methylation does not further disrupt DCC localization. In all panels DAPI is shown in grayscale and scale bar equals 5 µm. **B.** Quantification of colocalization by Pearson's correlation coefficient *R* indicating the degree of overlap between the DCC and X-Paint masks. *R* measurements were binned every 0.1 from 0 to 1 (where 0 indicates no detectable overlap and 1 indicates perfect colocalization) and percent of total sample per bin is shown. See Figure S7 for statistical analysis.

Colocalization analysis in the *htz-1* mutant background revealed that the degree of DCC mislocalization is unchanged upon Set1/MLL depletion (Fig. 6). A majority of the colocalization values collected in wildtype treated with empty vector RNAi are above 0.5 (16/18) and the average *R* is 0.61. The average *R* value in *htz-1* homozygous animas treated with vector control RNAi is significantly lower than wildtype (0.47). The average *R* (0.43–0.48) and profiles for Set1/MLL depletion are very similar to empty vector control RNAi in the *htz-1* homozygous background. We used one-way ANOVA analysis to determine whether there was any statistical difference among the colocalization data collected. When comparing the control and Set1/MLL RNAi samples in the *htz-1* background, no statistical difference was observed. However, addition of the control RNAi in wildtype data indicated a significant difference (*F* = 4.29, *F_Crit_* = 2.30, *P* = 0.0014). By Tukey's test, the significant differences in colocalization are found between the wildtype control and each of the *htz-1* background samples and not between the *htz-1* control and the Set1/MLL RNAi samples in the *htz-1* background (Fig. S7). We conclude that depletion of H3K4 methylation does not enhance the DCC mislocalization defect observed in *htz-1* mutants.

### Set1/MLL homologs do not function in dosage compensation

To confirm that changes in H3K4 methylation do not influence dosage compensation function, we utilized a genetic assay to test whether Set1/MLL complex subunits contribute genetically to dosage compensation. In this sensitized genetic assay we deplete components of the Set1/MLL complexes by RNAi in the *him-8 xol-1 sex-1* mutant background [4], [23]. The *him-8* mutation results in an average 38% male progeny. However, the *xol-1* mutation turns on dosage compensation and all male progeny die as a result [82]. The *sex-1* mutation weakens dosage compensation function, thereby sensitizing the assay [83]. Therefore, RNAi depletion of a gene required for dosage compensation function will rescue some of the males in this background.

**Figure 7:**
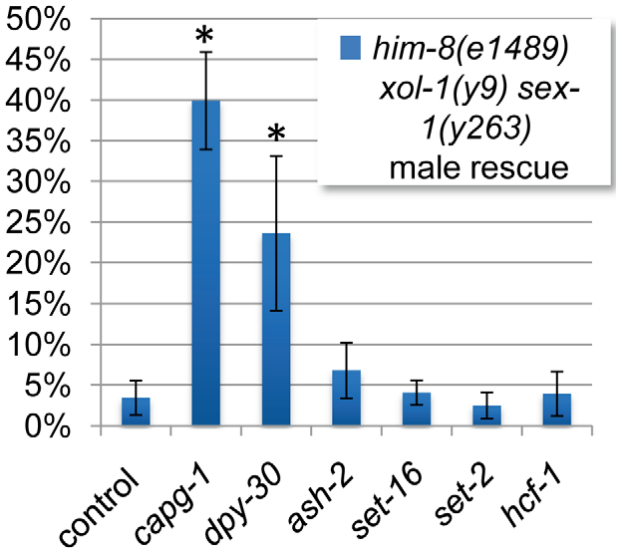
H3K4 methylation compexes do not function in dosage compensation. Rescue of sensitized male animals in which the *xol-1* mutation has ectopically turned on dosage compensation is used as a measure of genetic function of dosage compensation. Depletion of DCC subunit CAPG-1 by RNAi is able to disrupt ectopic dosage compensation, rescuing about 40% of expected male progeny. The only Set1/MLL complex subunit depletion with significant male rescue is *dpy-30*. Star indicates *p*<0.05 by T-test (*p* = 6.83×10^−5^ for *dpy-30* and *p* = 1.03×10^−9^ for *capg-1*).

The DCC component *capg-1* results in significant male rescue in this assay. However, the only Set1/MLL component with significant male rescue was *dpy-30* (*p* = 6.83×10^−5^ by t-test analysis) (Fig. 7). This suggests that *dpy-30* is unique among Set1/MLL components in its contribution to dosage compensation function. Combined with the data outlined above we conclude that the function of DPY-30 in dosage compensation is largely separate from its role in H3K4 methylation.

## Discussion

It has long been hypothesized that chromatin organization plays a substantial role in driving protein complexes to their proper targets within the genome. The predicted mechanism is that chromatin organization blocks access to all but a subset of potential binding sites at any given time. In support of this, functional transcription factor binding sites are commonly depleted of nucleosomes in human T-cells [27]. Also,in *C. elegans*, ectopic expression of the pharynx-specific transcription factor, PHA-4 in intestinal cells does not result in binding of PHA-4 to pharynx-specific genes, suggesting that these sites are unavailable for binding in the intestinal nuclei [84]. A survey of 21 transcription factor binding profiles in *Drosophila* has recently revealed that chromatin accessibility is a better predictor of target site occupancy than *in vitro* affinity [85]. Targeting of the X-chromosome specific DCC in *C. elegans* also involves both sequence and chromatin organization [17], [18], [20], [23].

Recently, it has become increasingly clear that in *C. elegans* the X and autosomal chromatin makeup is significantly different both at the population level by high resolution ChIP-chip and single-cell resolution microscopy studies [23], [64], [86], [87]. HTZ-1 was the first reported chromatin component found to be differentially incorporated on the dosage compensated X chromosomes [23], [29]. More recently, H4K20me1 and H3K27me1 were found at higher levels on X and it was suggested that this may be functionally important for gene regulation on the X chromosomes [64]. We have found that the dosage compensated X chromosomes have reduced levels of H3K4me3, similar to what has been reported for HTZ-1, and slightly higher levels of H3K4me1. Depletion of H3K4me3 and HTZ-1 are both independent of dosage compensation, but enrichment of monomethylated H3K4 may be subject to regulation by dosage compensation. The fact that loss of dosage compensation function in *sdc-1* animals abolishes enrichment of H3K4me1 suggests that the transcriptional regulation function of dosage compensation may involve H3K4me1. However, the fact that we do not observe any changes in DCC localization in the Set1/MLL depletion animals with lowest H3K4me1 signal (*set-2* or *set-16(RNAi)* in *set-2* mutant animals, see Fig. 5), suggests that if there is a role for monomethylation of H3K4, it is likely downstream of DCC localization. We did not detect any genetic function of either *set-2* or *set-16* in dosage compensation, suggesting that the overall contribution of H3K4me1 to chromosome-wide gene regulation by the DCC may be minor. Alternatively, the function of an as-yet unidentified methyltranferase may be responsible for the enrichment of H3K4me1 on the X chromosomes and this methyltransferase may function in dosage compensation. This hypothesis could be tested in the future by looking for expression changes of dosage compensated genes in animals with reduced H3K4me1 and by testing other methyltransferase genes for function in dosage compensation.

An attractive hypothesis was that the depletions of HTZ-1 and H3K4me3 on the X chromosomes function as an X-chromatin signature utilized by dosage compensation to selectively bind the X in hermaphrodites. Further support for this hypothesis was the fact that the Set1/MLL component DPY-30 is required for both normal levels of H3K4me3, and is required for dosage compensation (see figures 3 and S2) [60]. However, the fact that we did not observe disruption of DCC localization or an enhancement of DCC mislocalization in *htz-1* upon depletion of H3K4me3 by Set1/MLL depletion or mutation suggests that this is unlikely (Figures 4, 5, 6). Additionally, the striking difference in DCC mislocalization phenotypes in *dpy-30* and *htz-1* mutant animals is consistent with these factors regulating DCC localization in distinct ways (Fig. 2).

Upon depletion or mutation of all of the Set1/MLL homologs in *C. elegans*, we observed effects on global methylation in a manner consistent with what is known about the complex function in previous work (Fig. 3, S1, S2, S5 and S6). DPY-30 actually has a less severe effect on overall methylation than does ASH-2 or the catalytic subunits SET-2 and SET-16 (Fig. S4). In spite of the more severe effect on methylation levels upon depletion of ASH-2, SET-2, and SET-16, we observed no effect on DCC localization. Additionally, the reduced H3K4me3 we observe in Set1/MLL complex depletion or mutant animals does not correlate with an effect on DCC binding to chromatin. Therefore, the role of DPY-30 in DCC targeting is likely to be independent of its activity in known H3K4 methyltransferase complexes. Finally, genetic analysis revealed no evidence for Set1/MLL function in dosage compensation (Fig. 7).

How does DPY-30 contribute to dosage compensation if not through the Set1/MLL complexes? Current evidence indicates a physical interaction between DPY-30 and the DCC recruitment protein SDC-2. DPY-30 immunoprecipitation pulls down SDC-2 (although a reciprocal interaction was not observed), and DPY-30 is found at DCC binding sites in an SDC-2 dependent manner [7]. The binding domains that have been mapped by structure-function analyses between Ash2L/DPY-30 in mammals and Bre2/Sdc1 in budding yeast [58] are not found in SDC-2 nor in any of the other known dosage compensation proteins by blast analysis (although the *C. elegans* ASH-2 is identified in these searches). The proposed interaction between SDC-2 and DPY-30, if direct, is likely through a domain entirely different from the ASH-2 interacting domain, or the interaction may be indirect, requiring an unidentified intermediate. The DPY-30 homolog in yeast, Sdc1, functions in the dimerization of the whole complex through interactions with Bre2 (ASH-2) and in homodimer formation [58], [59], [88]. DPY-30 may play a similar role within the DCC, aiding the assembly of the DCC onto SDC-2.

The unique DCC mislocalization phenotype of *dpy-30* animals suggests that DPY-30 plays a distinct role in dosage compensation. In *sdc-2* mutant adult nuclei, IF experiments revealed loss of the Condensin I^DC^ signal ([4] and unpublished results). Although Pferdehirt et al 2011 report that DPY-27 levels are unchanged in *sdc-2* embryos, the loss of signal in adults suggests that over the course of development, DCC proteins are lost. However, in *dpy-30* adults, the DCC, although grossly mislocalized, is still easily detectable. In *dpy-30* embryos, binding of DCC proteins DPY-27 and SDC-3 is nearly absent at sites involved in recruitment, identical to the effect of *sdc-2* mutation. It would be interesting to test how *dpy-30* affects SDC-2 levels and localization. It is possible that SDC-2 is specifically required for the long-term stability of the DCC as well as chromosome specific loading onto the X. In that case, if SDC-2 is not able to bind chromatin, its presence in the nucleus may still be able to perform its DCC stabilizing function. Then we would predict that *dpy-30*'s function in dosage compensation is to facilitate SDC-2-specific binding on chromatin such that loss of *dpy-30* results in failure of SDC-2 to “anchor” the stable complex to chromatin. The overall effect on complex localization would be specific loss of DCC at sites where SDC-2 is absolutely required for DCC binding. Alternatively, *sdc-*2 may be required for positively regulating expression of the other dosage compensation genes in addition to directing DCC loading onto the X. All DCC proteins except SDC-2 are maternally loaded into oocytes, which could account for the similarity in DPY-27 levels in *sdc-2* and wild-type embryos. Eventually this maternally provided DCC supply is lost in *sdc-2* animals. Whether *sdc-2* regulates DCC expression at the level of protein stability or transcription could be discerned by observation of mRNA and protein levels in developing *sdc-2* embryos. Understanding the nature of the DPY-30-SDC-2 interaction in dosage compensation will be the crucial first step in understanding how DPY-30 promotes DCC binding and may also shed light on novel Set1/MLL-independent functions of DPY-30 in other organisms.

## Materials and Methods

### Strains and alleles

All strains used were maintained as described [89]. Strains include N2 Bristol strain (wildtype); TY4403 *him-8(e1489)* IV; *xol-1(y9) sex-1(y263)* X; TY4161*sdc-1(e415)* and TY3936 *dpy-21(e428)*; TY1936 *dpy-30(y228)*/nT1[unc-?(n754dm)let-?];EKM11 *htz-1(tm2469)*IV/*nT1(qIs51)* IV,V; EKM32 *hcf-1(ok559)IV*; and EKM31 *set-2(ok952)III*. Null mutants *set-2(bn129)III* and *wdr-5.1(ok1417)III* (gifts of F.Palladino) were also used. *hcf-1(ok559)* and *set-2(ok952)* were back-crossed four times prior to analysis. Analysis of *dpy-30(y228)* and *htz-1(tm2469)* was performed in *m+z-* animals.

### RNA interference

*E. coli* HT115 bacteria expressing double stranded RNA for *capg-1*, *set-2,dpy-30, hcf-1,* F52B11.1 (CFP1 homolog), F21H12.1 (RbBP5 homolog), *wdr-5.1*, *swd-3.2, swd-3.3*, *swd-2.1*, and *swd-2.2* or vector control (polylinker), were used for feeding RNAi using the Ahringer feeding RNAi clones [90]. To generate RNAi vectors for *set-16* and the 3′ *ash-2* the regions were PCR amplified, digested with NotI and XhoI (*set-16*) or XhoI and SpeI (*ash-2*), and cloned into the DT7 vector as described [90]. The following primers were used for amplification:

*set-16*:

cgcggccgccgagcaacaacagggag and cctcgaggctgagaaggctgtttgc

*ash-2*:

gactcgaggctacacgttcacctgcaaa and tgactagtgccattcctttttctgcttg

RNAi experiments for IF/FISH and to score male rescue in *him-8(e1489) IV*; *xol-1(y9) sex-1(y263) X* were conducted as described [23]. Briefly, L1/L2 animals were grown on feeding RNAi plates at 20°C until the L4 stage. Five to ten L4 hermaphrodites were then moved onto fresh feeding RNAi plates for 24 hours at 20°C to throw progeny. Adult (P0) animals were burned off the next day and their progeny were grown at 20°C. For the male rescue assay the number of embryos laid was counted at this time. Male counts were performed 3 days later. The F1 progeny were analyzed for IF/FISH 24 hours post-L4 (young adults).

### Immunostaining

Adult animals were dissected and stained as described [19]. Millipore Anti-Monomethyl Histone H3 (Lys4) rabbit polyclonal antibody (cat # 07-436) was used at 1∶100 dilution. Abcam H3 dimethyl K4 goat polyclonal antibody (ab11946) was used at 1∶100. Millipore Anti-Trimethyl Histone H3 (Lys4) clone CMA304 mouse monoclonal antibody (Cat. #05-1339) was used at 1∶100. Upstate anti-trimethyl-Histone H3(Lys27) (Cat. #17-622) rabbit polyclonal was used at 1∶200 Affinity purified polyclonal rabbit anti-DPY-27 and polyclonal rat anti-CAPG-1 [4] were used at 1∶100. Polyclonal rabbit anti-DPY-28 (gift of K. Hagstrom) was used at 1∶500 and polyclonal rabbit anti-MIX-1 (gift of R. Chan) was used at 1∶100. Mouse monoclonal Anti-α-tubulin by Sigma (T6199) was used at 1∶500. Mouse monoclonal antibody [Mab414] to Nuclear Pore Complex Proteins by Abcam (ab24609) was used at 1∶1000. Secondary antibodies used are: Fluorescein (FITC) conjugated donkey α-rabbit (Jackson ImmunoResearch) and Cy3 conjugated donkey α-rabbit IgG (Jackson ImmunoResearch) both at a dilution of 1∶100. Images were captured with a Hamamatsu ORCA-ERGA CCD camera mounted on an Olympus BX61 motorized X-drive microscope using a 60X oil immersion objective. Captured images were deconvolved using 3i Slidebook imaging software. Projected images were taken at 0.2 µm intervals through samples. Adobe Photoshop was used for assembling images.

### Fluorescent *in situ* hybridization

FISH probe templates were generated by degenerate oligonucleotide primed PCR to amplify purified yeast artificial chromosome DNA. The labeled X-paint probe was prepared and used as described [19]. Hybridization was performed on young adult animals (24 hours post-L4) as described [23].

### Quantification of fluorescence levels and colocalization

3-D z-stack images of nuclei were collected as described above. 3i Slidebook imaging software was used to set masks of histone modification/variant localization (H3K4 me1, me2, me3, and HTZ-1) and DCC localization on the 3-D image (although 3-D projections are shown in Fig.1, projections were not used for quantification). The histone modification/variant mask was used to determine the average fluorescence intensity for the histone modification/variant in the whole nucleus. The DCC mask was used to determine the average fluorescence intensity for the histone modification/variant signal specifically on the X chromosomes. The ratio of histone modification/variant signal on X versus the nucleus as a whole was determined by dividing the average signal intensity in the DCC mask divided by the average signal intensity of the histone modification/variant mask.

3i Slidebook imaging software was used to measure colocalization of DPY-27 (FITC) and X-Paint (Cy3) signals on images obtained as described above. The FITC:Cy3 correlation coefficient was recorded and used as an indication of colocalization between DPY-27 and X-Paint as described [23].

### Detergent extraction

Detergent extraction of nucleoplasmic protein from dissected nuclei was performed by dissecting animals in 1X sperm salts plus 1% Triton detergent [91]. Dissected animals were then processed for Fluorescent *in situ* hybridization followed by immunofluorescence.

### Western blot analysis

100 (2X lanes) and 50 (1X lanes) young adult Set1RNAi worms (24 hours post-L4) were picked into 1XM9, washed, and incubated for ten minutes at 95°C in 19 µl SDS-PAGE loading dye (0.1 M Tris-HCl pH 6.8, 75 M Urea, 2% SDS, Bromophenol Blue for color) plus 1 µl β-mercaptoethanol. 10 ul of the worm extract in loading buffer was loaded per lane into a 15% polyacrylamide gel. SDS-PAGE was performed and protein was transferred onto nitrocellulose. The following antibodies and dilutions were used: Millipore Anti-Monomethyl Histone H3 (Lys4) rabbit polyclonal antibody at 1∶500, Abcam H3 dimethyl K4 goat polyclonal antibody at 1∶1000, Anti-Trimethyl Histone H3 (Lys4) clone CMA304 mouse monoclonal antibody at 1∶1000, and mouse monoclonal Anti-α-tubulin at 1∶1000. Secondary antibodies used were as follows: anti-mouse-HRP, anti-goat-HRP, and anti-rabbit-HRP (Jackson ImmunoResearch) all at 1∶5000.

## Supporting Information

Figure S1 **Depletion of Set1/MLL components reduces H3K4me3 but does not affect DCC binding.** H3K4me3 (green) and DPY-27 (red) IF in control RNAi and Set1/MLL depletion animals shows a reduced H3K4me3 signal upon Set1/MLL RNAi in adult, hermaphrodite intestinal nuclei. The names of the mammalian homologs are included for reference. In all panels DAPI is shown in grayscale and scale bar equals 5 µm.
(TIF)

Figure S2 **H3K4me1 and me2 in Set1/MLL depletion nuclei. A.** H3K4me1 (green) and CAPG-1 (red) co-staining in control and Set1/MLL depletion nuclei (adult intestine). Reduction in H3K4me1 is only observed in SET-2 and SET-16 depletion animals. **B.** H3K4me2 (green) and DPY-27 (red) co-staining slight reductions in signal are observed in all Set1/MLL depletion nuclei except in *dpy-30*. In all panels DAPI is shown in grayscale and scale bar equals 5 µm.
(TIF)

Figure S3 **H3K27me3 signal unchanged in Set1/MLL depletion and mutant adults.** H3K27me3 (green) and DPY-27 (red) co-staining in control and Set1/MLL depletion (**A**) and mutant (**B**) nuclei reveal no change in H3K27Me3 staining. In all panels DAPI is shown in grayscale and scale bar equals 5 µm.
(TIF)

Figure S4 **H3K4 methylation levels upon Set1/MLL depletion in wild-type, hermaphrodite adults.** Western blot analysis of H3K4 methylated species in young adult hermaphrodites (100 and 50 per lane). H3K4me1 is reduced in *set-2* and *set-16*. H3K4me2 levels are reduced in all except *dpy-30*. H3K4me3 levels are reduced in *set-2, set-16*, *ash-2*, and *dpy-30*. α-Tubulin is used as a loading control.
(TIF)

Figure S5 **H3K4 methylation in** ***set-2, hcf-1, and wdr-5.1*** **deletion mutants.** H3K4 methylation staining is in green, DPY-27 (with H3K4me2 and me3 co-stain) and CAPG-1 (with H3K4me1 co-stain) is shown in red. *hcf-1(ok559)* adult intestinal nuclei show reduced H3K4me3 staining, while *set-2(ok952),* a hypomorphic allele, *set-2(bn129)* and *wdr-5.1(ok1417)* both null alleles [81] show reductions in all marks to varying degrees. No DCC localization/binding phenotype is observed in these backgrounds. In all panels DAPI is shown in grayscale and scale bar equals 5 µm.
(TIF)

Figure S6 **H3K4me1 and me2 after Set1/MLL depletion in** ***set-2(ok952)*** **adults. A.** H3K4me1 (green) and CAPG-1 (red) IF and **B.** H3K4me2 (green) and DPY-27 IF in adult intestinal nuclei. Although effects on methylation can be detected, there is no effect on DCC localization in the *set-2* mutant and Set1/MLL depletion animals.
(TIF)

Figure S7 **Statistical analysis of X-Paint/DCC colocalization study in** ***htz-1(tm2469)*** **background.** One-way ANOVA of all colocalization data reveals statistically significant differences between samples. When the wildtype control data is omitted, the null is accepted. Tukey's test reveals that the significant differences between samples are specifically between the wildtype control and each of the *htz-1* (control and Set1/MLL depletion) samples.
(TIF)
