# Supplementary figures and images for: Regulation of DCC Localization by HTZ-1/H2A.Z and DPY-30 Does not Correlate with H3K4 Methylation Levels
Source: PLoS One. 2011 Oct 5;6(10):e25973. doi: 10.1371/journal.pone.0025973 (PMC3187824; doi:10.1371/journal.pone.0025973)

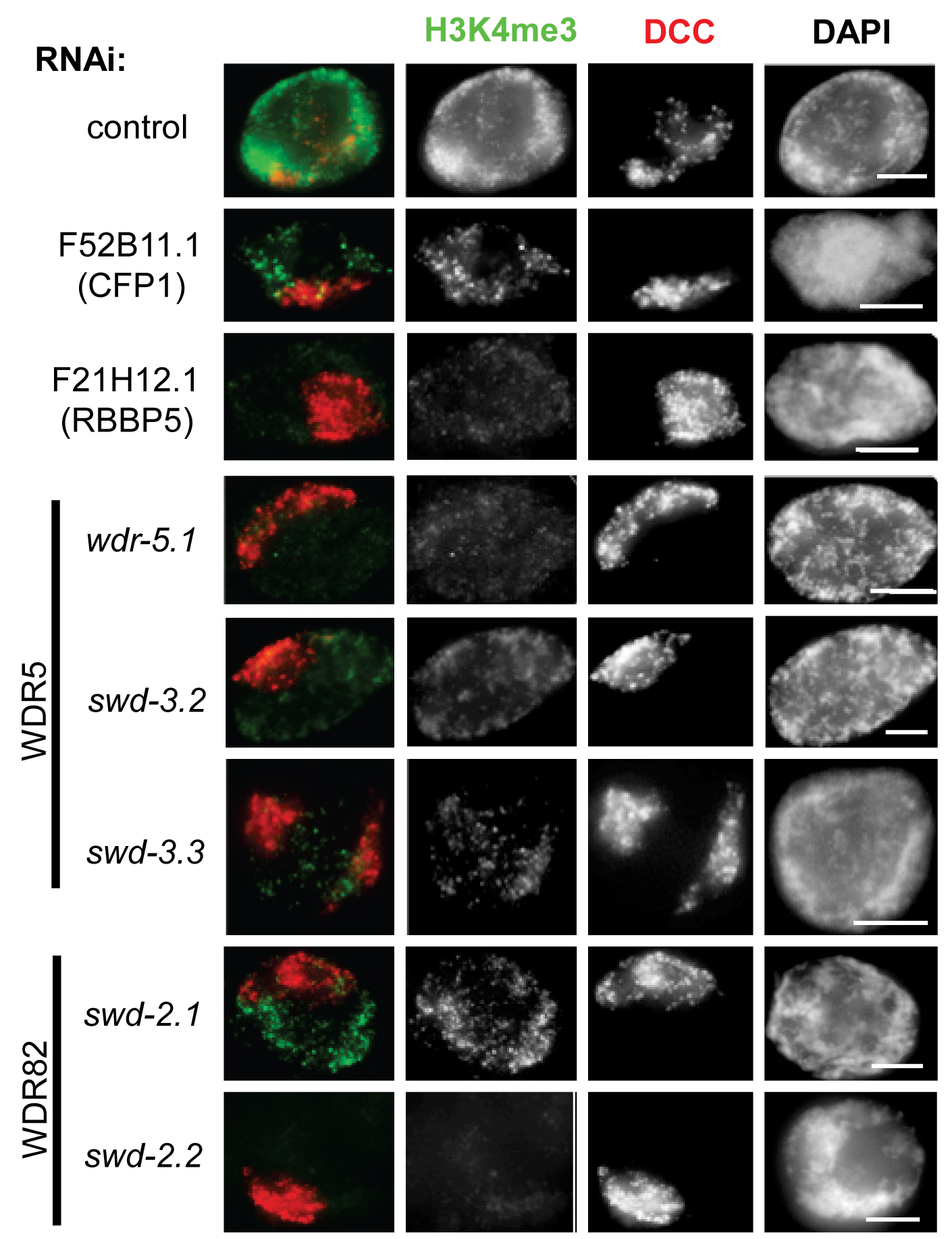

Supplement: Figure S1 — Depletion of Set1/MLL components reduces H3K4me3 but does not affect DCC binding. H3K4me3 (green) and DPY-27 (red) IF in control RNAi and Set1/MLL depletion animals shows a reduced H3K4me3 signal upon Set1/MLL RNAi in adult, hermaphrodite intestinal nuclei. The names of the mammalian homologs are included for reference. In all panels DAPI is shown in grayscale and scale bar equals 5 µm. (TIF) [file pone.0025973.s001.tif]

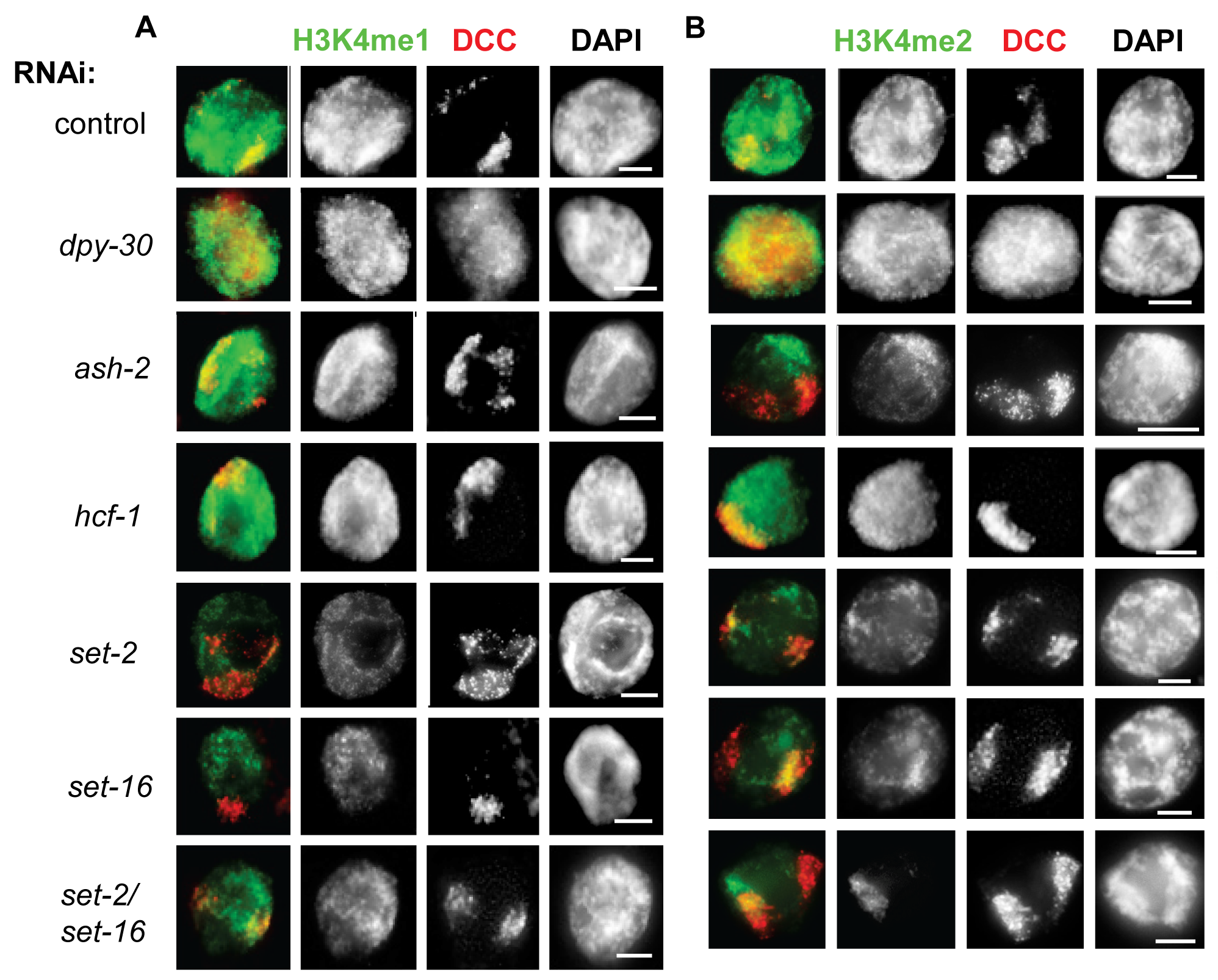

Supplement: Figure S2 — H3K4me1 and me2 in Set1/MLL depletion nuclei. A. H3K4me1 (green) and CAPG-1 (red) co-staining in control and Set1/MLL depletion nuclei (adult intestine). Reduction in H3K4me1 is only observed in SET-2 and SET-16 depletion animals. B. H3K4me2 (green) and DPY-27 (red) co-staining slight reductions in signal are observed in all Set1/MLL depletion nuclei except in dpy-30. In all panels DAPI is shown in grayscale and scale bar equals 5 µm. (TIF) [file pone.0025973.s002.tif]

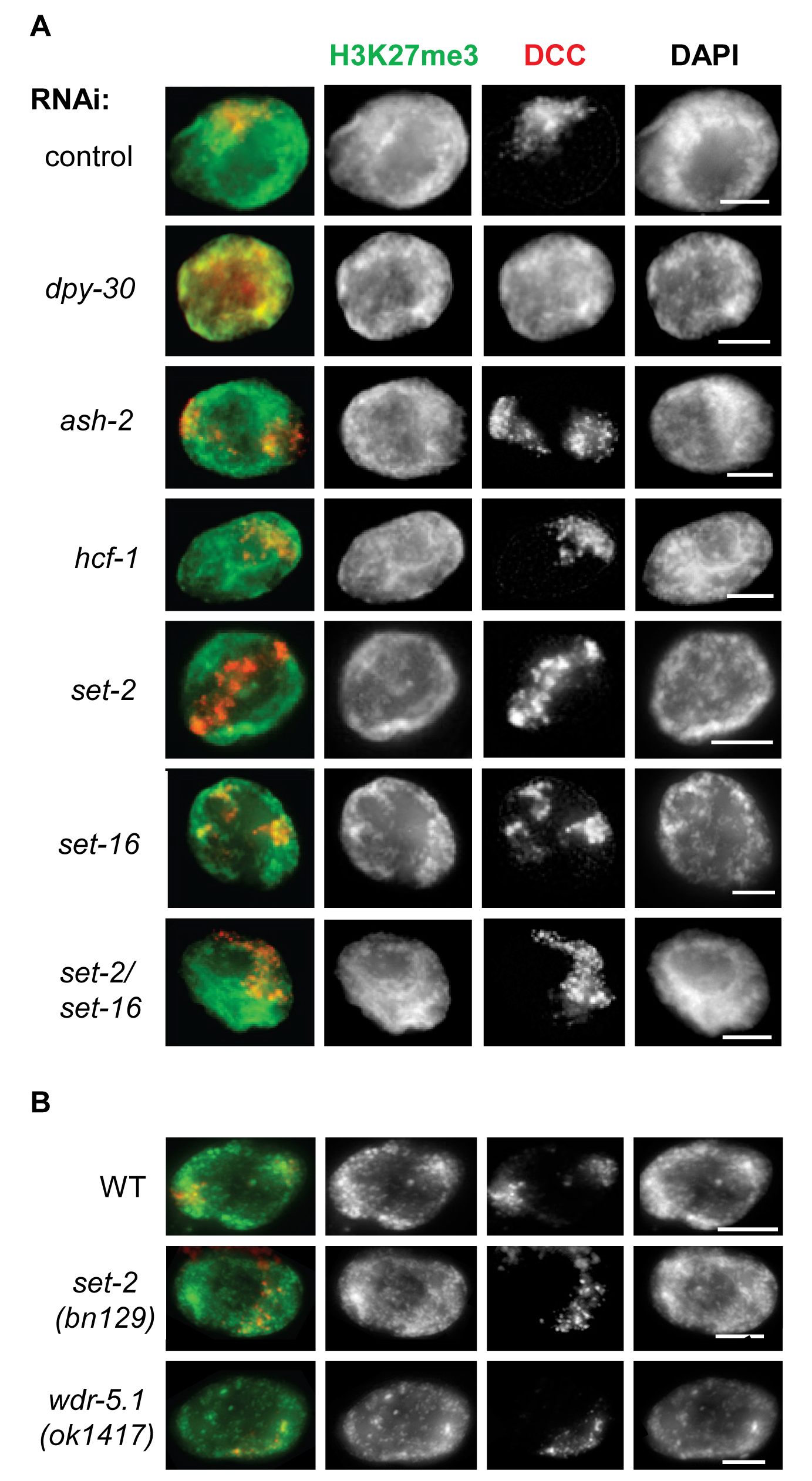

Supplement: Figure S3 — H3K27me3 signal unchanged in Set1/MLL depletion and mutant adults. H3K27me3 (green) and DPY-27 (red) co-staining in control and Set1/MLL depletion (A) and mutant (B) nuclei reveal no change in H3K27Me3 staining. In all panels DAPI is shown in grayscale and scale bar equals 5 µm. (TIF) [file pone.0025973.s003.tif]

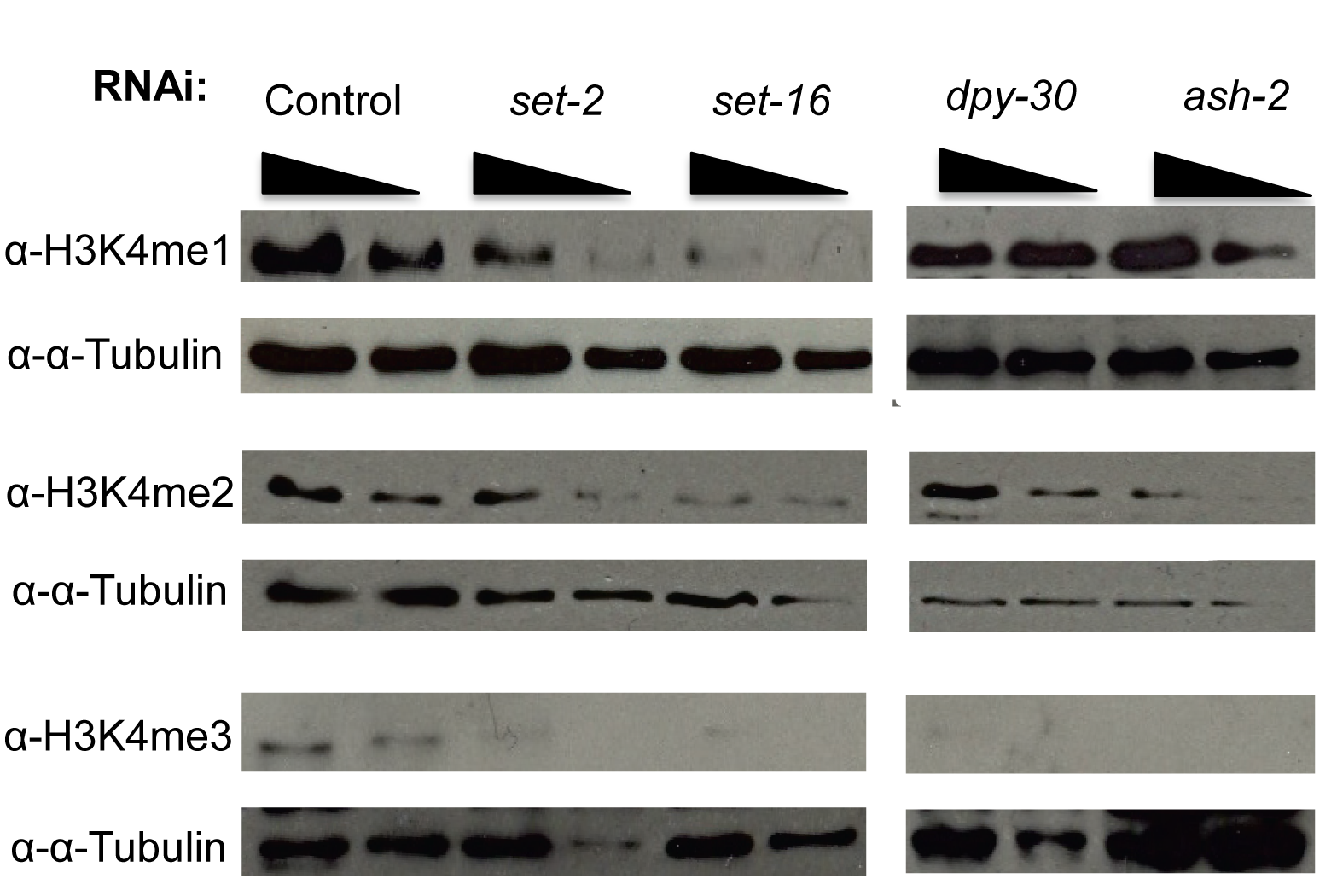

Supplement: Figure S4 — H3K4 methylation levels upon Set1/MLL depletion in wild-type, hermaphrodite adults. Western blot analysis of H3K4 methylated species in young adult hermaphrodites (100 and 50 per lane). H3K4me1 is reduced in set-2 and set-16. H3K4me2 levels are reduced in all except dpy-30. H3K4me3 levels are reduced in set-2, set-16, ash-2, and dpy-30. α-Tubulin is used as a loading control. (TIF) [file pone.0025973.s004.tif]

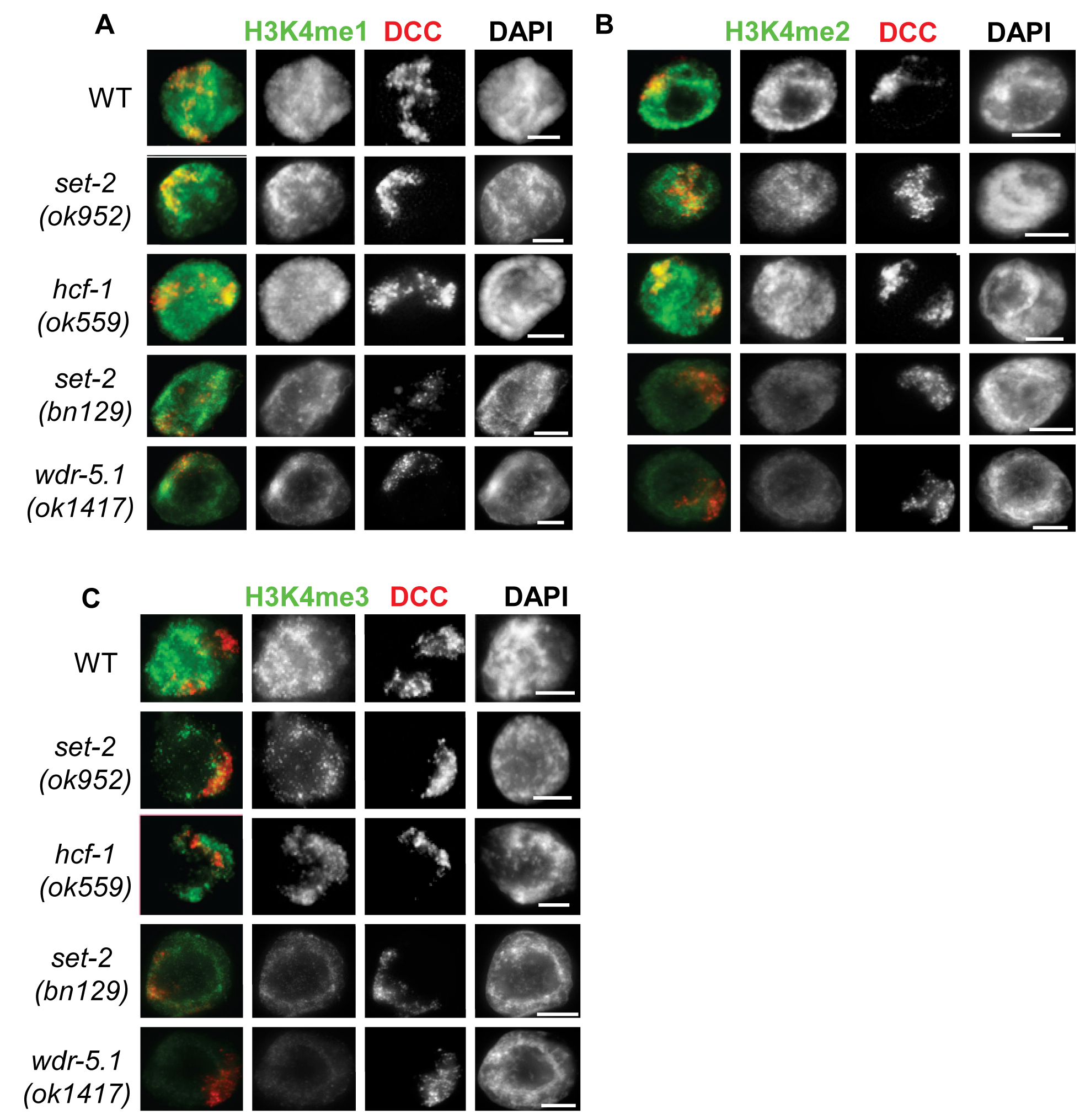

Supplement: Figure S5 — H3K4 methylation in set-2, hcf-1, and wdr-5.1 deletion mutants. H3K4 methylation staining is in green, DPY-27 (with H3K4me2 and me3 co-stain) and CAPG-1 (with H3K4me1 co-stain) is shown in red. hcf-1(ok559) adult intestinal nuclei show reduced H3K4me3 staining, while set-2(ok952), a hypomorphic allele, set-2(bn129) and wdr-5.1(ok1417) both null alleles [81] show reductions in all marks to varying degrees. No DCC localization/binding phenotype is observed in these backgrounds. In all panels DAPI is shown in grayscale and scale bar equals 5 µm. (TIF) [file pone.0025973.s005.tif]

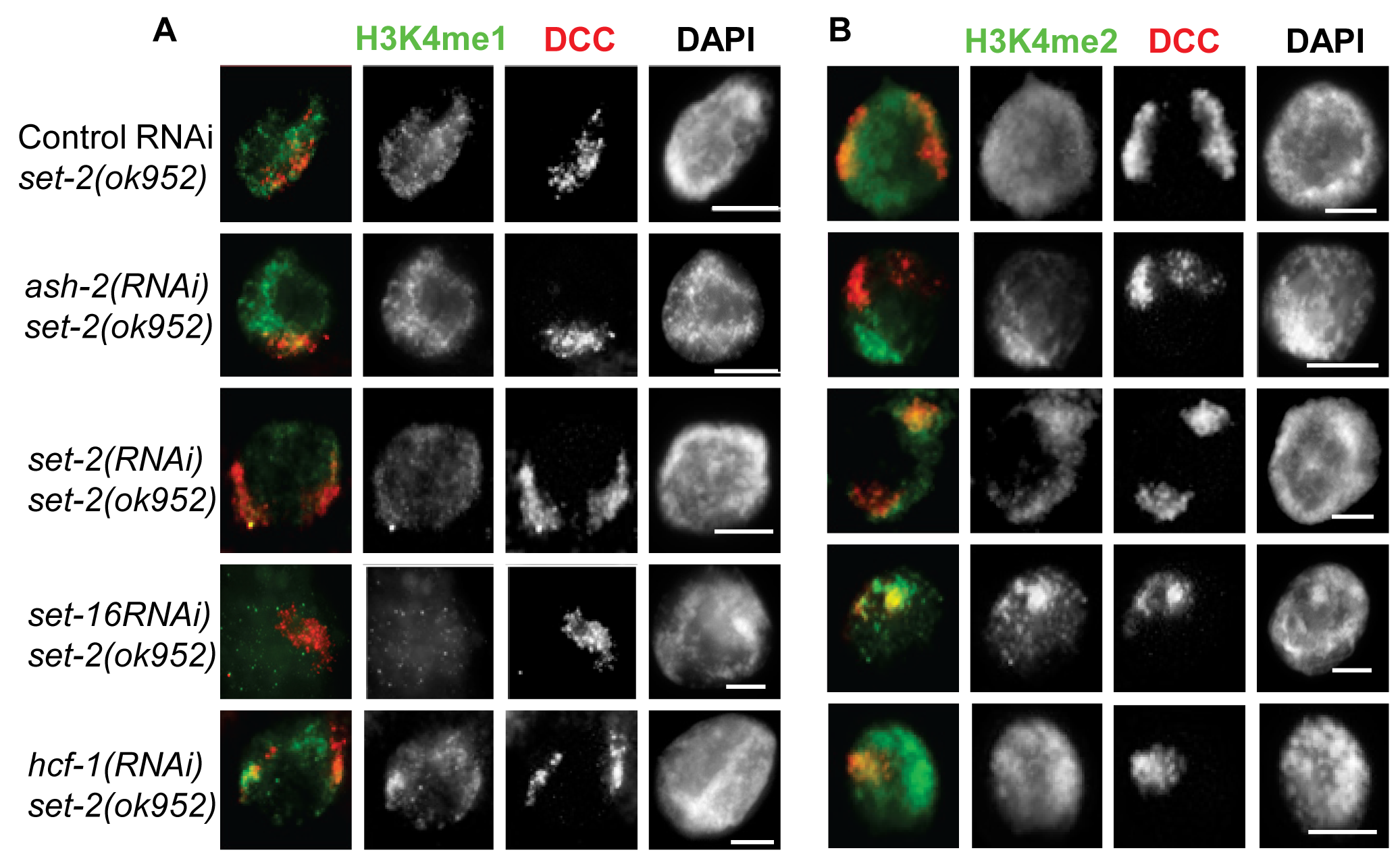

Supplement: Figure S6 — H3K4me1 and me2 after Set1/MLL depletion in set-2(ok952) adults. A. H3K4me1 (green) and CAPG-1 (red) IF and B. H3K4me2 (green) and DPY-27 IF in adult intestinal nuclei. Although effects on methylation can be detected, there is no effect on DCC localization in the set-2 mutant and Set1/MLL depletion animals. (TIF) [file pone.0025973.s006.tif]

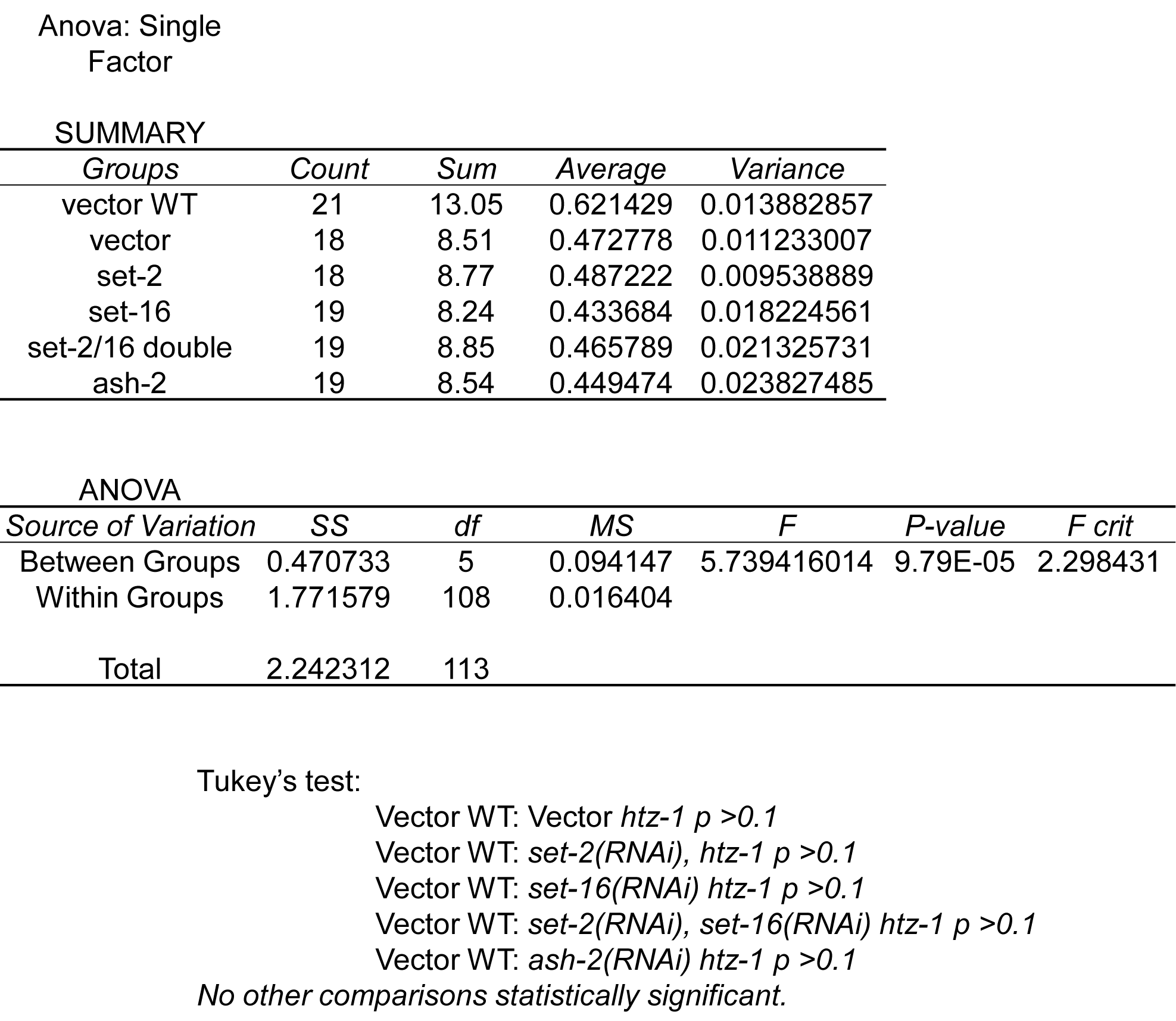

Supplement: Figure S7 — Statistical analysis of X-Paint/DCC colocalization study in htz-1(tm2469) background. One-way ANOVA of all colocalization data reveals statistically significant differences between samples. When the wildtype control data is omitted, the null is accepted. Tukey's test reveals that the significant differences between samples are specifically between the wildtype control and each of the htz-1 (control and Set1/MLL depletion) samples. (TIF) [file pone.0025973.s007.tif]
